# Techniques for mesoappendix transection and appendix resection: insights from the ESTES SnapAppy study

**DOI:** 10.1007/s00068-022-02191-8

**Published:** 2023-01-24

**Authors:** Gary Alan Bass, Lewis J. Kaplan, Maximilian Peter Forssten, Thomas N. Walsh, Yang Cao, Shahin Mohseni, ESTES SnapAppy Group, Rebecka Ahl Hulme, Alan Biloslavo, Hayato Kurihara, Isidro Martinez-Casas, Jorge Pereira, Arvid Pourlotfi, Éanna J. Ryan, Matti Tolonen, Nayef Louri, Fatema Nedham, Jamal Hashem, Martin Corbally, Abeer Farhan, Hamad Al Hamad, Rawan Elhennawy, Mariam AlKooheji, Manar AlYusuf, Wissal Aknouche, Anas A. Zeidan, Yusuf S. Alsaffar, Edgar Lipping, Peep Talving, Sten Saar, Katrina Graumann, Liis Kibuspuu, Eduard Harkov, Gisele Aaltonen, Iines S. Sillman, Sami Haapanen, Hanna Lampela, Henna Sammalkorpi, Sofia Eskola, Altti Laakso, Johan Back, Ulla Kettunen, Antti M. Nummi, Anika Szwedyc, Taina Nykänen, Rolle Rantala, Elisa J. Mäkäräinen-Uhlbäck, Sanna A. Meriläinen, Heikki I. Huhta, Jukka M. J. Rintala, Kirsi E. M. Laitakari, Elina Lietzen, Paulina Salminen, Risto K. A. Rapola, Vahid Zangouri, Mohammad Y. Karami, Sedigheh Tahmasebi, Majid Akrami, Alireza Golchini, Faranak Bahrami, Sean M. Johnston, Sean T. Lim, Irele Ifijeh Ahonkhai, Eltahir Eltagani, Odhran K. Ryan, Ailbhe O’Driscoll-Collins, Aine O’Neill, Zakiya Penny, Orlaith Kelly, Carolyn Cullinane, Ian Reynolds, Helen Heneghan, Sean Martin, Des Winter, Matthew Davey, Maha Alkhattab, Aoife J. Lowery, Michael J. Kerin, Aisling M. Hogan, Martin S. Davey, Ke En Oh, Syed Mohammad Umar Kabir, Huilun Huan, Charlotte Aziz, Michael Sugrue, Jessica M. Ryan, Tara M. Connelly, Mohammad Alhazmi, Youssef Al-Mukhaizeem, Fiachra Cooke, Peter M. Neary, Arnold D. K. Hill, Michael R. Boland, Angus J. Lloyd, Frances Fallon, Eoin F. Cleere, James Toale, Patrick A. Boland, Michael Devine, Conor Keady, Sarah Hunter, M. Kevin Barry, Michael E. Kelly, Aidan T. O’Dowling, Ben Creavin, Dara O. Kavanagh, Paul Neary, Paul F. Ridgway, Cathleen A. McCarrick, Jarlath Bolger, Barry Maguire, Cian Keogh, Surbhi Chawla, John Conneely, Emilie McCormack, Ben Shanahan, Nicola Raftery, Darragh Rice, Niall McInerney, Aine Stakelum, Jan Mares, Jonavan Tan, Mark Hanna, Ishwarya Balasubramanian, Christina Fleming, Guy Barsky, Gad Shaked, Simone Giudici, Martina Ceolin, Simona Mei, Francesca Mazzarella, Annalisa Zucca, Susanna Terranova, Nicolo de Manzini, Diego Visconti, Emanuele Doria, Mauro Santarelli, Giovanni Scotton, Francesca Notte, Giacomo Bertelli, Anna Malpaga, Giulia Armatura, Antonio Frena, Dario Tartaglia, Federico Coccolini, Camilla Cremonini, Enrico Cicuttin, Alessio Mazzoni, Massimo Chiarugi, Constança M. Azevedo, Filipa D. Mendes, Luis Q Faria, Carlos Nazario, Daniela Machado, Miguel Semiao, Carlos Casimiro, Jose Pinto, Tiago Pavão, Raquel Pereira, Bruno Barbosa, Nadia Tenreiro, Catia Ferreira, Goncalo Guidi, Daniela C. Martins, Clara Leal, Bruno B. Vieira, Luís S. Castro, Aldara Faria, Alberto Figueira, Mauro Sousa, Pedro Rodrigues, Rodrigo Roquette, Ricardo Ribeiro, Paulo Cardoso, Joana Domingues, Maria Isabel Manso, Rute Pereira, Tatiana Revez, Bogdan D. Dumbrava, Florin Turcu, Ionut Hutopila, Bogdana Banescu, Gerald Filip, Catalin Copaescu, Marcos Alba Valmorisco, Isabel Manzano Martín, Rocio Martín Garcíade de Arboleya, José Ortega Seda, Pablo Rodríguez González, Jose Antonio Becerra Toro, Enrique Rodríguez Lara, Jose Antonio González Minchón, Juan José Segura-Sampedro, Sebastián Jerí-McFarlane, Alejandro Gil-Catalán, Andrea Craus-Miguel, Laura Fernández-Vega, Xavier González-Argenté, Mercedes Estaire-Gómez, Borja Camacho Fernández-Pacheco, Rebeca Vitón-Herrero, Elisa Jimenez-Higuera, Alejandro Barbero, José M. Valverde, Enrique Colás-Ruiz, Maria del Mar Escales-Oliver, Olga Claramonte-Bellmunt, Marta Castro-Suárez, Naila Pagés-Valle, José Andrés Cifuentes-Ródenas, Marta Merayo Alvarez, Jose Luis Michi Campos, Luis Alejandro García González, Beatriz Carrasco Aguilera, Jaime Iturbe Menéndez, Jose Luis Rodicio Miravalles, Carmen Rodríguez Haro, Sara Núñez O’Sullivan, Mariana García Virosta, María Hernández O’Reilly, Izaskun Balciscueta-Coltell, Javier Lorenzo-Perez, Sonia Martinez-Alcaide, Susana Martinez-Ramos, Maria Sebastian-Fuertes, Laura Gomez-Romer, Maria M. Pelloni, Aida Cristina Rahy-Martín, Andrés Felipe Yepes-Cano, Julio Reguera-Rosal, Jose A. Lopez-Ruiz, Beatriz Marenco, Marina Retamar-Gentil, Estela Romero-Vargas, Angeles Gil-Olarte, Aitor Landaluce-Olavarria, Begoña Estraviz-Mateos, Jose-Mario De Francisco-Rios, Aitor Sainz-Lete, Ane Emaldi-Abasolo, Manolo Leon-Valarezo, Claudia C. Moreira Lopes, Aintzane Lizarazu Perez, Araceli Rodriguez Gonzalez, Iñigo Augusto Ponce, Ignacio Maria Goena Iglesias, Cristina González-Prado, Guillermo Cabriada, Beatriz López, Michelle C. Otero, Nerea Muñoz-Plaza, Alberto Palomo, Fernando Mendoza-Moreno, Manuel Díez-Alonso, Francisca García-Moreno-Nisa, Belén Matías-García, Enrique Ovejero-Merino, Ana Quiroga-Valcárcel, Luis Sánchez-Guillén, Inmaculada Oller-Navarro, Álvaro Soler-Silva, Antonio Francisco Sanchís-López, Francisco Blanco-Antona, Luis Muñoz-Bellvis, Jaime López-Sánchez, Sonsoles Garrosa-Muñoz, Beatriz Barón-Salvador, Juan Manuel Nieto-Arranz, Andrea Campos-Serra, Raquel Gràcia-Roman, Anna Muñoz-Campaña, Carla Zerpa-Martin, Andrea Torrecilla-Portoles, Tessa Landa, Virginia Durán Muñoz-Cruzado, Felipe Pareja-Ciuró, Daniel Aparicio-Sánchez, Eduardo Perea del Pozo, Antonio Jesús García-Moriana, Sandra Dios-Barbeito, Carlos García-Sánchez, Victor Turrado-Rodriguez, Roser Termes-Serra, Paula Gonzalez-Atienza, Xavier Morales-Sevillano, Alba Torroella, César Ginestà, Alfredo Escartín, Ferney Gomez, Ana Pinillos, Jaume Ortega, Guillermo Lopez, Eric Gutierrez, Estela Membrilla-Fernandez, Francisco Ocho-Segarra, Ana María González-Castillo, Amalia Pelegrina-Manzano, Juan Guzmán-Ahumada, Juan Jose Sancho-Insenser, María Lourdes García-Jiménez, Laura Castro-Diez, Manuel González-Bermúdez, Mónica Torres-Díaz, Carla Madarro Pena, Angélica Blanco Rodríguez, Dhanisha Trivedi, Souheil Reda, Hans Edvardsson, Lovisa Strömmer, Eva-Corina Caragounis, Karin Sillén, Sofia Warfvinge, Fredrik Bergstedt, Philip Enström, Harald Olsson, Anders Rosemar, Nathalie Young, Agnieszka Popowicz, Johanna Lerström, Johanna Jäderbo, Folke Hammarqvist, Hanna Zacharias, Maria B. Wikström, Anna Stene Hurtsén, Haytham Bayadsi, Emma Jansson, Nils Brunstrom, Ellen B. Malers, Per I. Loftås, Anders Möller, Elena Atanasova, Simone N. Zwicky, Beat Schnüriger, Olga Rutka, Arjun T. Kattakayam, Mushfique Alam, John V. Taylor, Andrei Mihailescu, Eszter T. Karip, Ehtisham Zeb, Adam O’Connor, Goran Pokusevski, Mansoor Khan, Charlotte Florance, Christie Swaminathan, Shameen Jaunoo, Mohammed Sajid, Caoimhe C. Duffy, John Rees, Mark J. Seamon, Niels D. Martin, Ian J. McCurry, Emily A. Vail, Bradford C. Bormann, Daniel C. Cullinane, Jaswin S. Sawhney, Jonathan Dreifus, Forest R. Sheppard, Raul Coimbra, Paul Albini, Sara Edwards

**Affiliations:** 1 Division of Traumatology, Surgical Critical Care and Emergency Surgery, Perelman School of Medicine at the University of Pennsylvania, Philadelphia, USA; 2 Division of Trauma and Emergency Surgery, Department of Surgery, Orebro University Hospital and School of Medical Sciences, Orebro University, 701 85 Orebro, Sweden; 3 Center for Perioperative Outcomes Research and Transformation (CPORT), University of Pennsylvania, Philadelphia, USA; 4 Leonard Davis Institute of Health Economics (LDI), University of Pennsylvania, Philadelphia, USA; 5 Corporal Michael Crescenz Veterans Affairs Medical Center, Philadelphia, USA; 6 Royal College of Surgeons in Ireland Medical University, Busaiteen, Bahrain; 7 Department of Clinical Epidemiology and Biostatistics, School of Medical Sciences, Orebro University, Orebro, Sweden

**Keywords:** Acute appendicitis, Observational cohort, Complications, Mesoappendix, Appendix base, Appendectomy, Surgical technique

## Abstract

**Introduction:**

Surgically managed appendicitis exhibits great heterogeneity in techniques for mesoappendix transection and appendix amputation from its base. It is unclear whether a particular surgical technique provides outcome benefit or reduces complications.

**Material and methods:**

We undertook a pre-specified subgroup analysis of all patients who underwent laparoscopic appendectomy at index admission during SnapAppy (ClinicalTrials.gov Registration: NCT04365491). We collected routine, anonymized observational data regarding surgical technique, patient demographics and indices of disease severity, without change to clinical care pathway or usual surgeon preference. Outcome measures of interest were the incidence of complications, unplanned reoperation, readmission, admission to the ICU, death, hospital length of stay, and procedure duration. We used Poisson regression models with robust standard errors to calculate incident rate ratios (IRRs) and 95% confidence intervals (CIs).

**Results:**

Three-thousand seven hundred sixty-eight consecutive adult patients, included from 71 centers in 14 countries, were followed up from date of admission for 90 days. The mesoappendix was divided hemostatically using electrocautery in 1564(69.4%) and an energy device in 688(30.5%). The appendix was amputated by division of its base between looped ligatures in 1379(37.0%), with a stapler in 1421(38.1%) and between clips in 929(24.9%). The technique for securely dividing the appendix at its base in acutely inflamed (AAST Grade 1) appendicitis was equally divided between division between looped ligatures, clips and stapled transection. However, the technique used differed in complicated appendicitis (AAST Grade 2 +) compared with uncomplicated (Grade 1), with a shift toward transection of the appendix base by stapler (58% vs. 38%; *p < *0.001). While no statistical difference in outcomes could be detected between different techniques for division of appendix base, decreased risk of any [adjusted IRR (95% CI): 0.58 (0.41–0.82), *p = *0.002] and severe [adjusted IRR (95% CI): 0.33 (0.11–0.96), *p = *0.045] complications could be detected when using energy devices.

**Conclusions:**

Safe mesoappendix transection and appendix resection are accomplished using heterogeneous techniques. Technique selection for both mesoappendix transection and appendix resection correlates with AAST grade. Higher grade led to more ultrasonic tissue transection and stapled appendix resection. Higher AAST appendicitis grade also correlated with infection-related complication occurrence. Despite the overall well-tolerated heterogeneity of approaches to acute appendicitis, increasing disease acuity or complexity appears to encourage homogeneity of intraoperative surgical technique toward advanced adjuncts.

## Introduction

Acute appendicitis is one of the most common abdominal emergencies requiring acute surgery. The incidence of appendicitis is estimated at 100, 105, and 151 per 100,000 person years in North America, Eastern Europe, and Western Europe, respectively [1]. Since its description by Fitz in 1886, clinical investigations underscore the need for prompt therapy of the non-perforated appendix [2]. The 1894 publication describing the muscle splitting incision standardized the operative exposure for appendectomy. Since then, mortality associated with acute appendicitis has been reduced to nearly 0.1% due to further improvements in medical and surgical management [3]. The surgical approach has evolved over the decades from Fitz’s laparotomy and McArthur’s ‘gridiron incision’ at McBurney’s point for targeted appendectomy to the minimally invasive procedures that sprang from Semm’s innovative ‘pelvikoscopie’ in 1982 [4, 5]. However, there remains some opacity regarding the most efficacious surgical technique for mesoappendix transection, and for management of the appendix base. Each of these is key operative elements of appendectomy regardless of open or minimally invasive approach.

Safe mesoappendix division may be achieved by mechanical means (clips, intracorporeal suture), electrocautery, of tissue sealing devices using ultrasonic or tissue sealing energy approaches. To date, no randomized control trial or large prospective cohort study has compared the bleeding complication profile between techniques. Similarly, appendix resection at its base may be achieved by sharp division between suture ligatures or clips, or linearly arrayed staples. Despite the length of time that these approaches have been used, it remains unclear whether one technique is superior regarding post-appendectomy infection including appendiceal stump leak and cecal fistula formation. These questions are more difficult to answer using randomized controlled trials as entry criteria often exclude patients in whom the techniques would be deployed during routine clinical care. Instead, a prospective non-randomized observational study of strictly controlled patient cohorts using time-bound patient accrual and multicenter assessment may be better suited to answer current care questions. Such studies are known as snapshot audits, and this exploration parses data from the SnapAppy cohort study to answer questions regarding mesoappendix and appendix management as a preplanned evaluation of the specifics of appendicitis management using laparoscopic techniques [6–8].

## Methods

### Protocol

We conducted a prospective, observational, non-randomized multicenter cohort study, using standardized published methodology [9], in line with a pre-specified protocol which was registered with ClinicalTrials.gov (Trial # NCT04365491). The study enrolled all consecutive patients admitted with acute appendicitis in a 90-day window between November 1, 2020, and May 28, 2021, and followed those patients for 90 days post-admission (up to August 31, 2021). The study complied with both the Strengthening the Reporting of Observational Studies in Epidemiology (STROBE) guidelines and the Declaration of Helsinki.

### Center eligibility

Any unit undertaking adult acute care surgery was eligible to register to enter patients into the study. No minimum case volume, or center-specific limitations were applied. The study protocol was disseminated to registered members of the European Society of Trauma and Emergency Surgery (ESTES) and through national surgical societies.

### Patient eligibility

All adult patients (over 15 years of age) admitted for acute appendicitis who underwent laparoscopic appendectomy during index admission were included in the current study. Appendicitis was graded using the AAST Anatomic Disease Severity grading system for emergency general surgery that provides a uniform method to assess disease severity for a variety of conditions, including acute appendicitis [10–12]. The grading system uses clinical, radiographic, operative, and pathologic criteria to assign an incrementing ordinal severity score of 1 (mild disease limited to the organ) to 5 (widespread severe disease).

### Data capture

Data were recorded contemporaneously and stored on a secure, user-encrypted online platform (SMARTTrial^®^) without patient-identifiable information. Centers were asked to validate that all eligible patients during the study period had been entered, and to attain > 95% completeness of data field entry prior to final submission. The database was closed for analysis on October 1, 2021. Quality assurance guidance to ensure data fidelity was provided by at least one consultant/attending-level surgeon at each site.

### Outcome measures

The primary outcome measure was any postoperative complication within 30 days. Secondary outcomes were severe complications within 30 days defined as Clavien–Dindo classification grade 3 to 5 (reoperation, reintervention, unplanned admission to intensive care unit, organ support requirement, or death), length of stay (LOS), and procedure duration (PD) in minutes.

### Statistical analysis

**Fig. 1:**
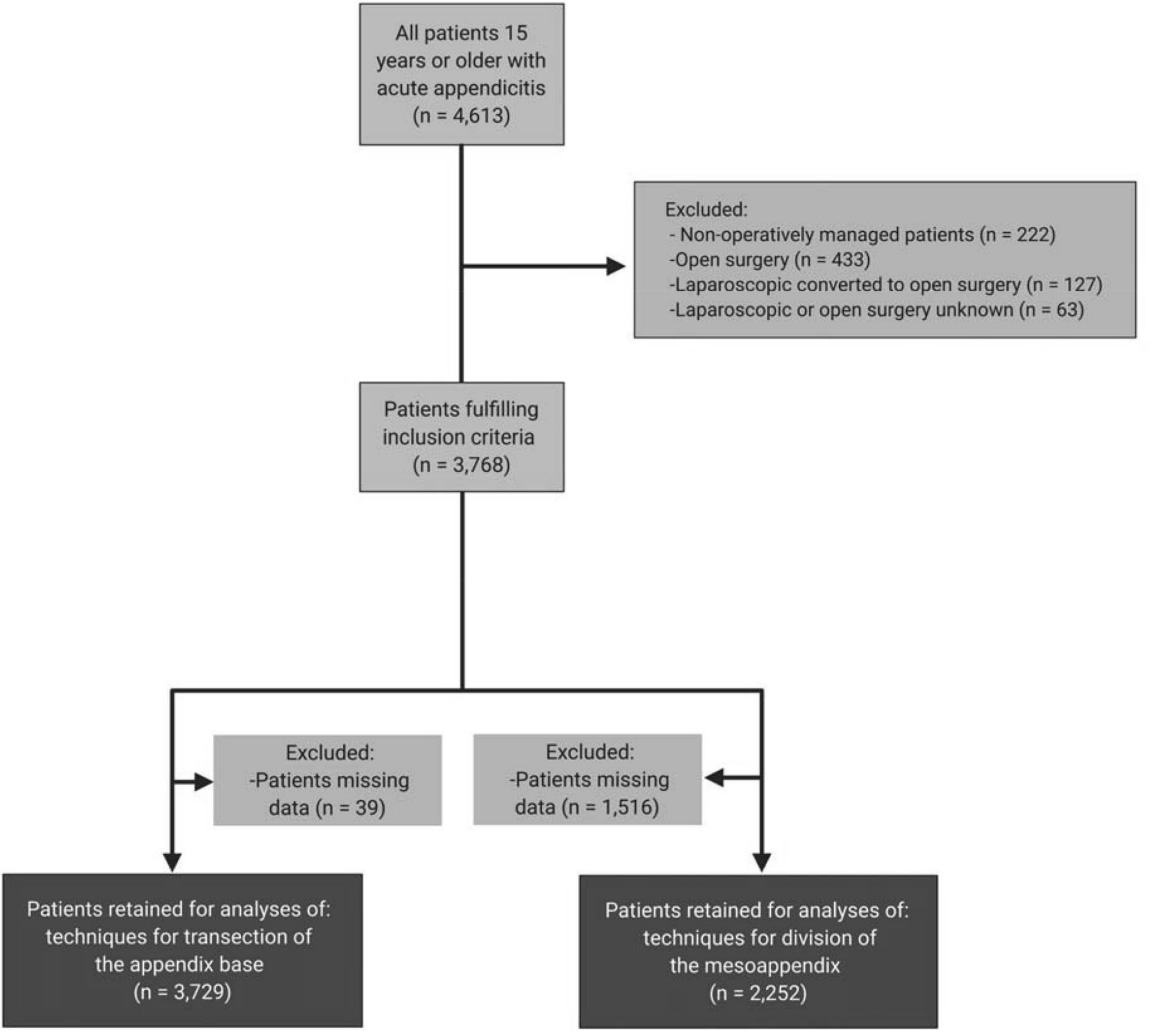
Study flow diagram illustrating the study patient denominator, sequential patient exclusions, and the ultimate numerator for patients included in final analyses

Patients who underwent a laparoscopic appendectomy were included for analysis. Patients who were missing data related to the technique used for mesoappendix transection or appendix resection were excluded from the associated analyses (study flow diagram; Fig. 1).

Patients were grouped based on the technique used for transection of the mesoappendix (electrocautery or energy device) or resection of the appendix (loop ligature, stapled, clipped). Descriptive results are presented as means and standard deviations (SDs) for continuous, normally distributed variables, medians and interquartile ranges (IQRs) for non-normally distributed continuous variables, as well as counts and percentages for categorical variables. Continuous, normally distributed variables were compared using a Student’s *t*-test, while non-normally distributed variables were compared using the Mann–Whitney *U*-test. A Chi-square test or Fisher’s exact test was used for categorical variables, as appropriate.

The relationship between the technique used and complications was determined using Poisson regression models with robust standard errors. The dependent variable was either any complication or severe complications, while the independent variables were the surgical technique as well as the patient’s age, sex, American Society of Anesthesiologists (ASA) classification, a history of previous abdominal surgery, ischemic heart disease, insulin-dependent diabetes, congestive heart failure, chronic renal disease, current smoking status, immunosuppression, the American Association for the Surgery of Trauma (AAST) appendicitis grade, time to surgery from admission, white blood cell count on admission, neutrophil percent on admission, C-reactive protein level on admission, as well as the country where the surgery was performed. Results of the statistical analyses are presented as incident rate ratios (IRRs) and 95% confidence intervals (CIs).

The association between the technique used and PD as well as LOS was evaluated using a quantile regression model. The independent variables were the same as previously utilized to assess the relationship between technique and complications. Results are presented as the median change in PD or LOS along with 95% CIs. Separate analyses were performed for each outcome as well as the stage of the appendectomy being investigated.

Multiple imputation by chained equations (MICE) was used to treat missing data [13]. In all analyses, a two-tailed p-value of less than 0.05 was considered statistically significant. Analyses were conducted with the statistical software R 4.1.1 (R Foundation for Statistical Computing, Vienna, Austria) using the tidyverse, mice, lubridate, readxl, writexl, robustbase, and quantreg packages [14].

### Ethical considerations

All participating centers had Institutional Review Board approval or equivalent. No patient consent was sought since the current study was purely observational and did not impact patient care. All data were de-identified when uploaded to the secure study database.

## Results

### Participating centers

Following an open call for participation in May 2020, 71 centers across 14 countries (Bahrain, Estonia, Finland, Iran, Ireland, Israel, Italy, Portugal, Romania, Spain, Sweden, Switzerland, UK, and USA) completed the local ethics approval process and proceeded to prospectively enroll patients.

### Comparison of surgical techniques for management of the appendix base

A total of 3729 patients were included in the analyses of the technique used for dividing the appendix base (Fig. 1). Histopathology revealed acute inflammation of the appendix in 90.4%, a normal appendix in 3.5% and neoplasm in 1.5%. The appendix was reportedly not sent for histopathologic evaluation in 4.5%, and data were missing or incomplete in 0.1%. Compared to patients whose appendix base was looped, those who had their appendix base divided using staples or clips were slightly older (33 vs. 38 and 35 years, *p < *0.001), had a marginally higher BMI (25.8 vs. 27.2 and 26.8, *p = *0.036), and were less fit for surgery according to their ASA classification (ASA ≥ 3: 5.1% vs. 10.6% and 7.7%, *p < *0.001). These patients were also more likely to have had their diagnosis confirmed using a computerized tomography (CT) scan (48.7% vs 66.2% and 55.1%, *p < *0.001). Compared to patients whose appendix base was looped or clipped, those who had their appendix base divided using staples were also more likely to have a perforated appendix (AAST grade ≥ 3: 15% vs 6.9% and 6.5%, *p < *0.001) (Table 1).

**Table 1:** Demographics, clinical characteristics, and crude outcomes grouped according to the technique used to divide the base of the appendix

	Looped (*N = *1379)	Staple (*N = *1,421)	Clips (*N = *929)	*p* value
Age, median [IQR]	33 [23–45]	38 [26–54]	35 [25–49]	< 0.001
Sex, *n* (%)				0.098
Female	665 (48.2)	629 (44.3)	424 (45.6)	
Male	711 (51.6)	791 (55.7)	503 (54.1)	
Missing	3 (0.2)	1 (0.1)	2 (0.2)	
Body mass index, mean (SD)	25.8 (± 5.2)	27.2 (± 8.5)	26.8 (± 22.9)	0.036
ASA classification, *n* (%)				< 0.001
1	920 (66.7)	791 (55.7)	581 (62.5)	
2	370 (26.8)	476 (33.5)	276 (29.7)	
3	67 (4.9)	139 (9.8)	69 (7.4)	
4	3 (0.2)	11 (0.8)	3 (0.3)	
Missing	19 (1.4)	4 (0.3)	0 (0.0)	
Duration of symptoms, *n* (%)				< 0.001
< 12 h	232 (16.8)	210 (14.8)	178 (19.2)	
12–24 h	436 (31.6)	478 (33.6)	345 (37.1)	
24–48 h	378 (27.4)	338 (23.8)	196 (21.1)	
48–72 h	152 (11.0)	168 (11.8)	101 (10.9)	
72–96 h	87 (6.3)	91 (6.4)	43 (4.6)	
> 96 h	76 (5.5)	128 (9.0)	55 (5.9)	
Missing	18 (1.3)	8 (0.6)	11 (1.2)	
AAST severity, *n* (%)				< 0.001
Grade 1: acutely inflamed appendix; intact	783 (56.8)	792 (55.7)	533 (57.4)	
Grade 2: gangrenous appendix; intact	33 (2.4)	94 (6.6)	36 (3.9)	
Grade 3: perforated appendix with local contamination	58 (4.2)	122 (8.6)	40 (4.3)	
Grade 4: perforated appendix with phlegmon/abscess	32 (2.3)	79 (5.6)	18 (1.9)	
Grade 5: perforated appendix with generalized peritonitis	5 (0.4)	11 (0.8)	3 (0.3)	
Missing	468 (33.9)	323 (22.7)	299 (32.2)	
Procedure duration, mean (SD)	59.5 (± 36.5)	64.9 (± 28.9)	51.1 (± 22.5)	< 0.001
Missing	68 (4.9)	23 (1.6)	27 (2.9)	
Length of stay, median [IQR]	2.0 [1.5–3.0]	2.0 [1.2–3.7]	1.5 [0.96–2.1]	< 0.001
Missing	55 (4.0)	37 (2.6)	18 (1.9)	
Complications within 30 days, *n* (%)	183 (13.3)	206 (14.5)	81 (8.7)	< 0.001
Wound infection	11 (0.8)	22 (1.5)	13 (1.4)	0.170
Wound dehiscence	4 (0.3)	12 (0.8)	3 (0.3)	0.077
Pelvic abscess	32 (2.3)	59 (4.2)	23 (2.5)	0.009
Subphrenic abscess	2 (0.1)	3 (0.2)	0 (0.0)	0.448
Hemorrhage	2 (0.1)	4 (0.3)	4 (0.4)	0.426
Sepsis	5 (0.4)	9 (0.6)	7 (0.8)	0.423
Ileus	16 (1.2)	57 (4.0)	9 (1.0)	< 0.001
Other complication	124 (9.0)	83 (5.8)	42 (4.5)	< 0.001
Missing	0 (0.0)	5 (0.4)	0 (0.0)	
Severe complications within 30 days, *n* (%)	17 (1.2)	52 (3.7)	18 (1.9)	< 0.001
Missing	95 (6.9)	32 (2.3)	18 (1.9)	
Complication severity according to Clavien–Dindo classification, *n* (%)				< 0.001
None	1196 (86.7)	1215 (85.5)	848 (91.3)	
1	28 (2.0)	64 (4.5)	19 (2.0)	
2	43 (3.1)	58 (4.1)	26 (2.8)	
3a	9 (0.7)	31 (2.2)	10 (1.1)	
3b	8 (0.6)	19 (1.3)	7 (0.8)	
4a	0 (0.0)	1 (0.1)	0 (0.0)	
4b	0 (0.0)	0 (0.0)	0 (0.0)	
5	0 (0.0)	1 (0.1)	1 (0.1)	
Missing	95 (6.9)	32 (2.3)	18 (1.9)	
Reoperation, *n* (%)	15 (1.1)	22 (1.5)	10 (1.1)	0.465
Missing	18 (1.3)	13 (0.9)	3 (0.3)	
Readmission, *n* (%)	18 (1.3)	13 (0.9)	18 (1.9)	0.104

Temperature is measured in degrees Celsius. Length of stay is measured in days. Procedure duration is measured in days. A severe complication is defined as a Clavien–Dindo classification ≥ 3a

*ASA* American Society of Anesthesiologists; *WBC* white blood cell count; *CRP* C-reactive protein; *SIRS* systemic inflammatory response syndrome; *CT* computed tomography; *AAST* American Association for the Surgery of Trauma

Temperature is measured in degrees Celsius. Length of stay is measured in days. Procedure duration is measured in days. A severe complication is defined as a Clavien–Dindo classification ≥ 3a

The appendix was amputated by division from its base using looped ligatures in 1379 (37.0%), staples in 1421 (38.1%) and clips in 929 (24.9%). The technique for transection of the appendix at its base in acutely inflamed (AAST Grade 1) appendicitis was relatively equally divided between division between looped ligatures (37%), clips (25%) and stapler (38%). However, the technique used differed in complicated appendicitis (AAST Grade 2 +) migrated toward transection of the appendix base by stapler (58%) vs. loops (24%) or clips (18%) (*p < *0.001) (Table 1).

**Fig. 2:**
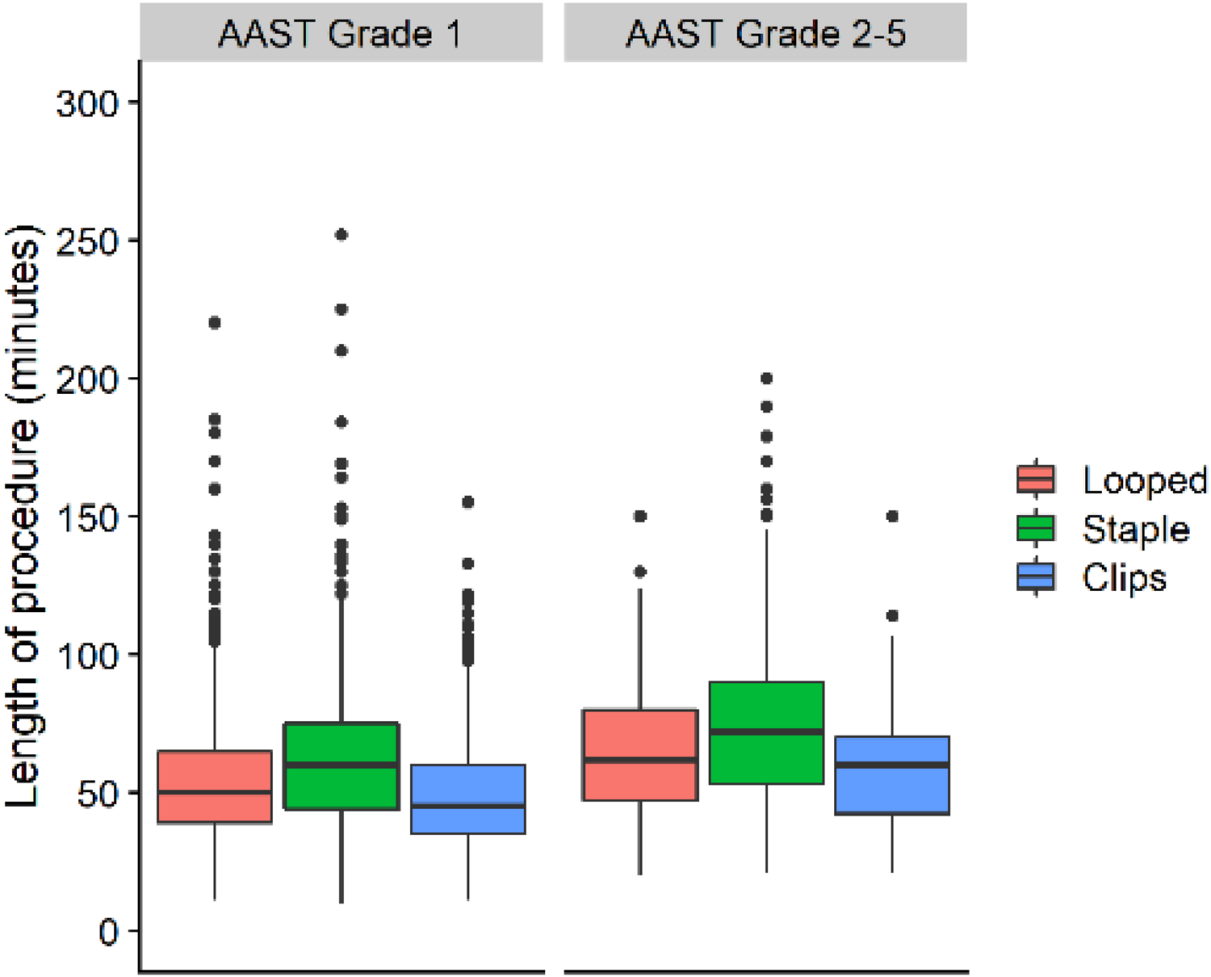
Neither procedure duration (a surrogate for case complexity), nor the utilization of advanced surgical instruments to seal and divide the appendix at its base, increased with AAST anatomic severity grade in patients undergoing laparoscopic appendectomy

Compared to patients whose appendix base was looped, overall procedural duration was greater for those who had their appendix base divided using staples (65 min vs 60 min, *p < *0.001), while it was shorter for those who had their appendix base divided using clips (51 min vs 60 min, *p < *0.001) (Fig. 2). The overall crude rate of any complication within 30 days was also marginally higher for those who had their appendix base divided using staples (14.5% vs 13.3%, *p < *0.001) and lower for those who had their appendix base divided using clips (8.7% vs 13.3%, *p < *0.001), compared to those who had their appendix base looped. However, patients who received staples or clips both had a higher crude rate of severe complications (3.7% and 1.9% vs 1.2%, *p < *0.001). There was no statistically significant difference in the crude rate of reoperations within the first 90 postoperative days (Table 1). After adjusting for potential confounders, there was no statistically significant difference in the risk of any [IRR (95% CI) 1.20 (0.94–1.53) and IRR (95% CI) 0.79 (0.57–1.08)] or severe complications [IRR (95% CI) 1.79 (0.97–3.31) and 1.09 (0.49–2.42)], when comparing the use of staples and clips with having the appendix base looped (Table 2). Similarly, while the median LOS was longer for patients who received staples [median LOS (95% CI): 0.26 days (0.14–0.37), *p < *0.001] and shorter for patients who received clips [median LOS (95% CI): − 0.16 days (− 0.08 to 0.25), *p < *0.001], compared to patients who had their appendix base looped, this difference was not clinically significant (Table 3). The median PD in patients who had their appendix base divided using stapler device was statistically significantly longer PD [median PD (95% CI): 5.75 min (3.35–8.15), *p < *0.001] compared to patients who had their appendix base looped or clipped (Table 4); however, this difference in PD holds no clinical significance.

**Table 2:** Incidence rate ratios (IRR) for postoperative complications after an appendectomy, according to the technique used to divide the base of the appendix and the mesoappendix

	IRR (95% CI)	*p* value
Any complication		
Appendix base division		
Looped	Ref	
Staple	1.20 (0.94–1.53)	0.139
Clips	0.79 (0.57–1.08)	0.142
Mesoappendix division		
Electrocautery	Ref	
Energy device	0.58 (0.41–0.82)	0.002
Severe complication		
Appendix base division		
Looped	Ref	
Staple	1.79 (0.97–3.31)	0.067
Clips	1.09 (0.49–2.42)	0.842
Mesoappendix division		
Electrocautery	Ref	
Energy device	0.33 (0.11–0.96)	0.045

Poisson regression models with robust standard errors. Multiple imputation with chained equations was used to manage missing values. The models are adjusted for age, sex, American Society of Anesthesiologists classification, a history of previous abdominal surgery, ischemic heart disease, insulin-dependent diabetes, congestive heart failure, chronic renal disease, current smoking status, immunosuppression, the American Association for the Surgery of Trauma appendicitis grade, time to surgery from admission, white blood cell count on admission, neutrophil percent on admission, C-reactive protein level on admission, as well as the country where the surgery was performed

**Table 3:** Change in median postoperative length of stay (days) after an appendectomy, according to the technique used to divide the base of the appendix and the mesoappendix

	Change in median length of stay (95% CI)	*p* value
Appendix base transection		
Looped	Ref	
Staple	0.26 (0.14–0.37)	< 0.001
Clips	− 0.16 (− 0.25 to 0.08)	< 0.001
Mesoappendix division		
Electrocautery	Ref	
Energy device	0.12 (− 0.03 to 0.26)	0.111

Length of stay is measured in days. Quantile regression model. Multiple imputation with chained equations was used to manage missing values. The models are adjusted for age, sex, American Society of Anesthesiologists classification, a history of previous abdominal surgery, ischemic heart disease, insulin-dependent diabetes, congestive heart failure, chronic renal disease, current smoking status, immunosuppression, the American Association for the Surgery of Trauma appendicitis grade, time to surgery from admission, white blood cell count on admission, neutrophil percent on admission, C-reactive protein level on admission, as well as the country where the surgery was performed

**Table 4:** Change in median appendectomy procedure duration (PD) in minutes, according to the technique used to divide the base of the appendix and the mesoappendix

	Change in median procedure duration (95% CI)	*p* value
Appendix base division		
Looped	Ref	
Staple	5.75 (3.35–8.15)	< 0.001
Clips	− 1.26 (− 3.47 to 0.96)	0.266
Mesoappendix division		
Electrocautery	Ref	
Energy device	− 0.05 (− 3.35 to 3.25)	0.976

Procedure duration is measured in minutes. Quantile regression model. Multiple imputation with chained equations was used to manage missing values. The models are adjusted for age, sex, American Society of Anesthesiologists classification, a history of previous abdominal surgery, ischemic heart disease, insulin-dependent diabetes, congestive heart failure, chronic renal disease, current smoking status, immunosuppression, the American Association for the Surgery of Trauma appendicitis grade, time to surgery from admission, white blood cell count on admission, neutrophil percent on admission, C-reactive protein level on admission, as well as the country where the surgery was performed

### Comparison of surgical techniques for management of the mesoappendix

A total of 2252 patients were included in the analyses of the technique used for dividing the mesoappendix (Fig. 1). The mesoappendix was divided using electrocautery in 1564 (69.4%) patients and an ultrasonic energy device in 688 (30.5%) and by other means (e.g., stapled or between clips or ligatures) in 1371(31.3%). Compared to patients who had their appendix divided using electrocautery, patients where an energy device was used instead were marginally older (37 vs 35, *p = *0.047) and less fit for surgery according to their ASA classification (ASA ≥ 3: 10.3% vs 7.4%, *p = *0.018). Energy device usage was also more prevalent in patients with perforation (AAST grade ≥ 3: 12.9% vs 9%, *p = *0.038) (Table 5).

**Table 5:** Demographics, clinical characteristics, and crude outcomes grouped according to the technique used to divide the mesoappendix

	Electrocautery (*N = *1564)	Energy device (*N = *688)	*p* value
Age, median [IQR]	35 [25–49]	37 [24–54]	0.047
Sex, *n* (%)			0.102
Female	742 (47.4)	300 (43.6)	
Male	820 (52.4)	387 (56.2)	
Missing	2 (0.1)	1 (0.1)	
Body mass index, mean (SD)	26.0 (± 5.3)	26.9 (± 9.3)	0.030
Missing	494 (31.6)	99 (14.4)	
ASA classification, *n* (%)			0.018
1	1002 (64.1)	400 (58.1)	
2	426 (27.2)	214 (31.1)	
3	108 (6.9)	67 (9.7)	
4	8 (0.5)	4 (0.6)	
Missing	20 (1.3)	3 (0.4)	
AAST severity, *n* (%)			0.038
Grade 1: acutely inflamed appendix; intact	817 (52.2)	370 (53.8)	
Grade 2: gangrenous appendix; intact	65 (4.2)	47 (6.8)	
Grade 3: perforated appendix with local contamination	78 (5.0)	52 (7.6)	
Grade 4: perforated appendix with phlegmon/abscess	54 (3.5)	34 (4.9)	
Grade 5: perforated appendix with generalized peritonitis	8 (0.5)	3 (0.4)	
Missing	542 (34.7)	182 (26.5)	
Length of stay, median [IQR]	1.8 [1.1–2.8]	2.1 [1.4–3.7]	< 0.001
Missing	33 (2.1)	34 (4.9)	
Complications within 30 days, *n* (%)	231 (14.8)	84 (12.2)	0.122
Wound infection	25 (1.6)	10 (1.5)	0.942
Wound dehiscence	8 (0.5)	5 (0.7)	0.750
Pelvic abscess	58 (3.7)	22 (3.2)	0.630
Subphrenic abscess	1 (0.1)	2 (0.3)	0.223
Hemorrhage	5 (0.3)	0 (0.0)	0.318
Sepsis	7 (0.4)	5 (0.7)	0.601
Ileus	43 (2.7)	11 (1.6)	0.135
Other complication	131 (8.4)	41 (6.0)	0.057
Missing	1 (0.1)	0 (0.0)	
Severe complications within 30 days, *n* (%)	47 (3.0)	14 (2.0)	0.231
Missing	73 (4.7)	26 (3.8)	
Reoperation, *n* (%)	24 (1.5)	10 (1.5)	1.00
Missing	14 (0.9)	5 (0.7)	
Readmission, *n* (%)	23 (1.5)	7 (1.0)	0.506

Temperature is measured in degrees Celsius. Length of stay is measured in days. Procedure duration is measured in days. A severe complication is defined as a Clavien–Dindo classification ≥ 3a

*ASA* American Society of Anesthesiologists; *WBC* white blood cell count; *CRP* C-reactive protein; *SIRS* systemic inflammatory response syndrome; *CT* computed tomography; *AAST* American Association for the Surgery of Trauma

**Fig. 3:**
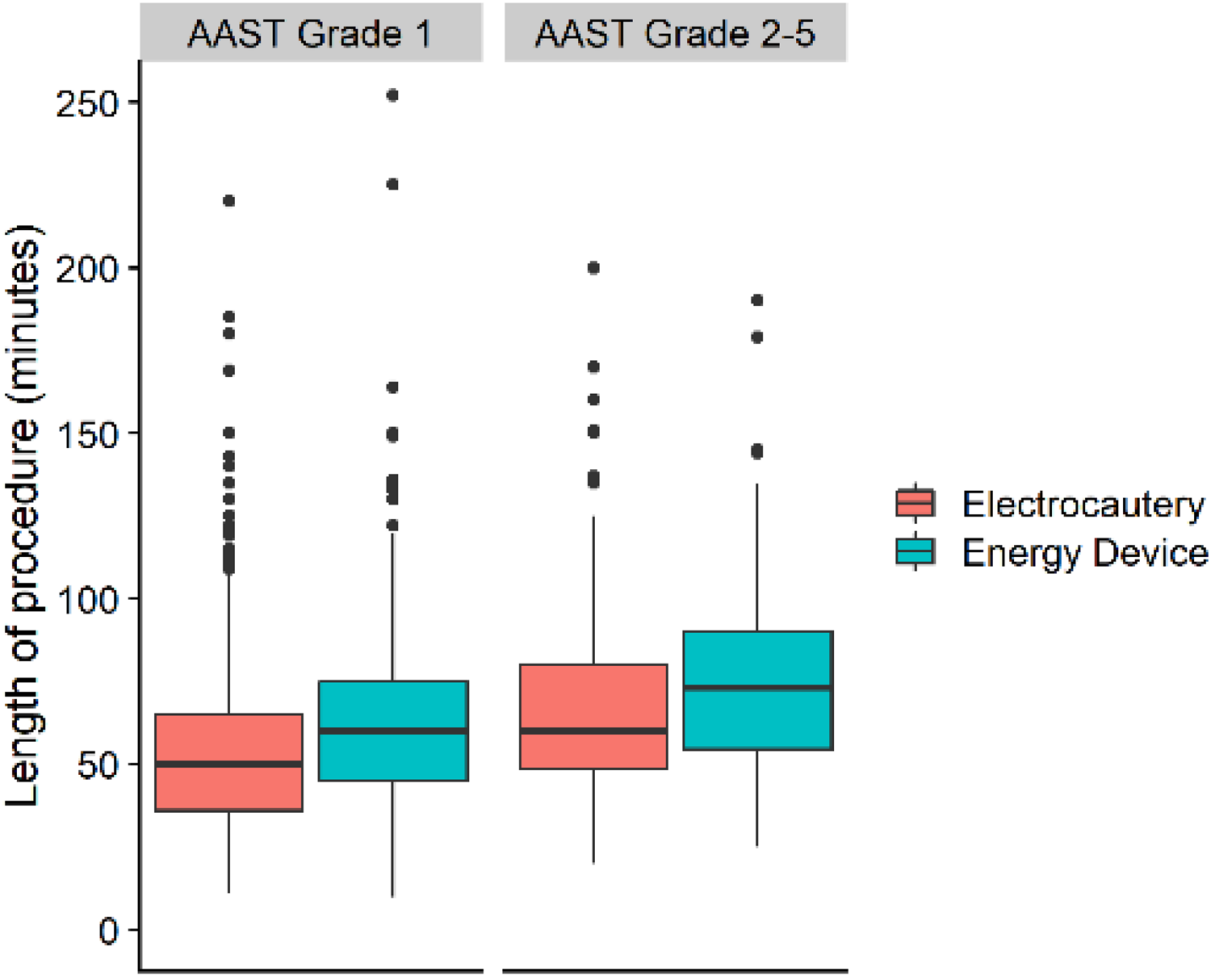
Procedure duration (a surrogate for case complexity) and the utilization of advanced surgical instruments to divide the mesoappendix, increased with AAST anatomic severity grade in patients undergoing laparoscopic appendectomy. Electrocautery devices include hook and Maryland dissectors connected to a current generator and can provide cutting or coagulation at the instrument tip. Ultrasonic or tissue sealing energy devices (including Ligasure and Harmonic Scalpel)

Patients who had their mesoappendix divided using an energy device both had an overall longer PD (65 min vs 57 min, *p < *0.001) and LOS (2.1 days vs 1.8 days, *p < *0.001). There were no statistically significant differences in the overall crude rate of any complications, severe complications, or reoperations (Table 5, Fig. 3). However, after adjusting for confounders in the regression analyses, energy devices were associated with a lower rate of any [adjusted IRR (95% CI): 0.58 (0.41–0.82), *p = *0.002] and severe [adjusted IRR (95% CI): 0.33 (0.11–0.96), *p = *0.045] complications (Table 2). The differences present in the crude PD and LOS were not statistically significant after adjusting for confounders in the regression analyses (Tables 3 and 4).

### Specific complications

**Fig. 4:**
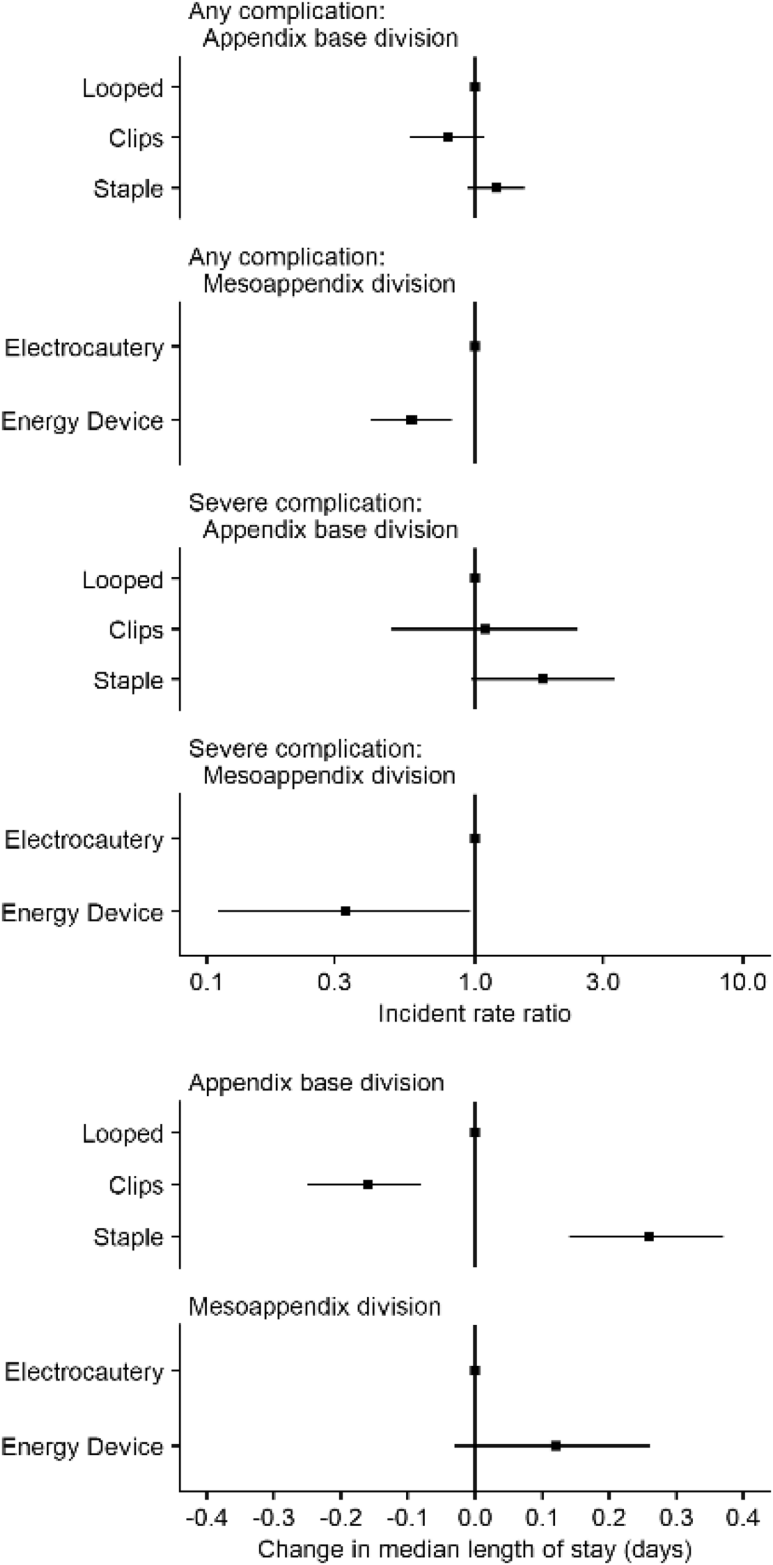
Forest plot of quantile regression

Postoperative complications within 30 days were reported in 12.6% of patients in the appendix base management cohort. The data-entry completion rate was high, with just 5 patients (0.1%) where data were unavailable. Infection-related complications accounted for 35% of all complications—surgical site infection was present in 46 patients (1.2%), postoperative organ-space infection was seen as pelvic abscess in 114 patients (3.1%), or subphrenic abscess in 5 patients (0.1%). Percutaneous interventional radiologic drainage of a postoperative intraperitoneal abscess was performed in 38 patients (1%). Postoperative ileus was seen in 82 patients (2.1%) and postoperative hemorrhage in 10 patients (0.2%). Other unspecified complications were recorded in 249 patients (6.7%). Forty-seven patients (1.3%) underwent re-operation during index admission (Fig. 4).

Postoperative complications within 30 days were reported in 315 (13.9%) of patients in the mesoappendix management cohort. Hemorrhagic complications were noted in 5 (0.2%) patients operated using electrocautery, versus 0 (0.0%) patients operated using an ultrasonic device (*p = *0.318). Infection-related complications were noted in 84 (5.4%) cases using electrocautery, versus 34 (4.9%) using an ultrasonic device (*p = *0.750). Severe complications (Clavien–Dindo 3 or above) within 30 days of operation were developed in 47 (3.0%) patients managed using electrocautery versus 14 (2.0%) patients managed using an ultrasonic device; (*p = *0.231).

## Discussion

Performance improvement in acute care surgery requires continuous re-evaluation of diagnostics, operative decision-making, and perioperative care. Nonetheless, contemporary appendicitis management exhibits great heterogeneity despite the frequency with which appendicitis is managed either operatively, or non-operatively. Indeed, several recent prospective randomized control trials both in Europe and the USA have demonstrated non-inferiority of antimicrobial-based pharmacologic management of early-stage acute appendicitis compared with appendectomy [15–19]. While these results have been met with great interest, and have provided therapeutic alternatives in the appropriate patient, the incidence of recurrent acute appendicitis, principally in patients with an appendicolith, ranging from 24 to 31%, is seen by some as a failure of non-operative management [20, 21]. Thus, outcomes related to surgical removal of the appendix remain of great importance for surgeons, Emergency Medicine clinicians, Primary Care physicians, as well as patients and their families.

Since outcomes are often related to condition stage or severity, a uniform manner by which to assess a process allows comparison across sites. The AAST disease severity grading system provides a mechanism to account for disease burden when performing comparative effectiveness research in emergency general surgery (EGS), including appendicitis [22, 23]. Increasing AAST grade for acute appendicitis is associated with increasing cost, complication rate, operative duration, length of stay, and need for open surgical technique in a variety of populations [22–25]. These data suggest that intraoperative assessment of appendicitis severity appears to impact surgical technique by influencing instrumentation selection. Energy device-driven mesoappendix transection and stapler-based appendix resection predominated as AAST appendicitis grade increased in comparison with all other approaches. It is intuitively attractive to link these two approaches with the notion of improved performance compared to electrocautery, clips, or loop ligatures. Nonetheless, other high-grade Clavien–Dindo classification complications were more common in those who underwent stapled appendix resection, before adjusting for confounders, reinforcing the notion that intraoperative tissue characteristics, anesthesia tolerance, or pre-operative assessment of comorbidities influenced instrument selection.

Instrument cost for frequent procedures such as laparoscopic appendectomy is important consideration in the OR and hospital budget assessments. Quantifying the total direct and indirect costs associated with an individual patient’s operation and hospital care is complex and multifactorial. A variety of factors impact the total assessment including but not limited to OR occupancy time, surgeon and anesthesiologist professional fees, pharmaceutical costs, equipment and supplies and personnel for room cleaning, reusable medical equipment acquisition and reprocessing/resterilization costs and single-use instrument acquisition costs (e.g., energy and stapling devices, loop ligatures, clips), acute care facility room costs, nursing costs, laboratory fees, and radiology tests as well as professional interpretations fees. Accordingly, instrumentation is only a small part of the total cost of operation related hospital care. Given the global heterogeneity in costs as well as patient charges, we did not undertake a cost analysis that would likely have been less fruitful than desired. Unfortunately, while surgeons are aware of their professional fees, many are less well informed regarding the costs of care such as those related to laboratory studies, general floor bed as opposed to ICU bed fees, or the cost of routine equipment or supplies. On the other hand, surgeons are acutely aware of postoperative complications and their impact on patient outcomes [26, 27].

In some healthcare systems, surgeon outcomes are publicly reportable. Importantly, many of the comparative assessment metrics are not acuity adjusted, nor coupled with other metrics of the acute care facility such as the case mix index. Additionally, completion of voluntary surveys may be freighted with subjectivity, as well as potentially overrepresenting those dissatisfied with care as they may be more likely to devote the required time. Therefore, postoperative complications may drive a skewed assessment of the surgeon when those assessments exist at a remove from an explanatory context.

Relatedly, the incidence of postoperative complications is a key quality metric for evaluating surgeon and hospital performance [28–35]. In over 4000 consecutive patients with acute appendicitis, the overall 90-day incidence of postoperative complications following appendectomy was 12.6%. Placing this in the context of existing literature (comprising retrospective administrative data and prospective randomized and non-randomized efficacy studies) [24–31] demonstrated divergent reporting practices as well as definitions used to identify complications. Many relevant retrospective contemporary studies reported an overall complication rate, with few defining complications in their methodology, or reporting the incidence of specific complications [24–31]. Total serious morbidity-modified National Surgical Quality Improvement Program (NSQIP) events were documented in 3.5% of patients randomized to surgical intervention in the 2020 Comparison of Outcomes of Antibiotic Drugs and Appendectomy (CODA) Trial. In 2015, the overall (not just serious) complication rate was 20.5% in the Finnish Antibiotic Therapy vs Appendectomy for Treatment of Uncomplicated Acute Appendicitis (APPAC) study [15, 20, 36]. An aggregated incidence of postoperative complications in a mixed population including uncomplicated and complicated appendicitis of 18.4% was derived from a meta-analysis of eleven trials assessing 1288 patients [37]. Therefore, our aggregate data align well with other studies—as does our specific major complication data—supporting the assertion that the captured audit data appropriately reflect outcomes in an unselected patient population that received contemporary acute appendicitis management.

Whether the untoward outcomes identified in our audit were specifically related to patient comorbidities, stage of presentation, surgical technique, instrumentation, or a combination of all four elements is unable to be parsed from the data. However, that geography did not directly link to a specific complication, nor a set of complications, argue that existing influencers were operative across all study sites, surgeons, and patients. Furthermore, the notion that emergency general surgery (EGS) patient outcomes are distinctly identifiable from those of elective patients regarding complication incidence and impact is a key point. Nascent efforts to establish an EGS database and morbidity and mortality review process metrics, morbidity and mortality calculators that assess comorbidity interaction rather than simple presence, as well as the growth of the AAST’s Acute Care Surgery fellowship programs underscores the differences that separate elective and emergency surgery patients. Rigorous evaluation of complications using a single data dictionary is likely to increase complication recognition and reporting in this unique patient group. It will be essential to frame the increased reporting in the context of condition severity, comorbidity, and acuity of intervention instead of simple presentation as an event frequency.

### Study limitations

Our data are supported by the strength of a time-bound prospective observational approach to a common condition managed across 14 countries, but nonetheless demonstrates important limitations. First, we did not secure long-term outcome data. In evaluating causality, or the impact of a trialed intervention compared to a control measure, long-term outcome data are key. This study was instead targeted at capturing an environmental scan of current practice and immediate outcomes to inform hypothesis generation. Moreover, the patients in this audit are all part of the “control” arm as they reflected usual care at each institution. Second, we did not assess the impact of ERAS protocols (if any were used), the use of nasogastric drainage, time to oral intake, the duration of postoperative antibiotic therapy, or the occurrence of multi-drug resistant organisms in those with infection-related complications. These are questions that have been assessed by other studies and would have expanded the collected data set without enhancing evaluation of the specifically targeted outcomes. Third, neither insurance status, socioeconomic group, nor ethnicity was assessed due to the wide variability across the 14 countries that was unified only by focusing on those undergoing laparoscopic appendectomy and their immediate outcomes. Assessments of community care, return to gainful employment, capability of completing activities of daily living and related elements was beyond the scope of this specific audit. While we present a large contemporary dataset, our analyses, and the conclusions drawn from them, are limited by the completeness of the data available (Fig. 1). We did not capture instances where surgeons chose to use a combination of approaches, nor did we capture the complex considerations around laparoscopic port size, design, and placement. We acknowledge this potential shortcoming in our data collection instrument design. The insights provided from these data would provide an interesting study of surgical ergonomics, as well as providing information on the potential impact of these equipment choices on procedure duration and rates of complication. The anticipated heterogeneity in port sizes, blunt versus sharp trocar design, reusable versus single-use, medical device manufacturer, modes of initial entry and insufflation, laparoscopic cameras, etc., used across 71 centers in 14 countries would greatly reduce power and strongly hamper any assumptions of association. This inquiry into efficacy would perhaps be best achieved by a different methodology, perhaps a well-designed tightly controlled prospective randomized control trial.

## Conclusions

Heterogeneity exists in the surgical techniques used to safely transect the mesoappendix and resect the appendix. Despite different instrumentation, operative time was remarkably similar. Instrument selection (energy devices and stapler) became more homogeneous with increasing AAST severity grade. This study’s findings may inform questions to be assessed that explore the specifics of intraoperative decision-making regarding instrumentation, as well as specific interventions to reduce the frequency of identified postoperative complications unrelated to the technical aspects of mesoappendix transection or appendix resection.

## Data Availability

The ESTES SnapAppy Group welcomes the use of these de-identified pooled data for further research that benefits patients. Requests can be submitted to the ESTES Research Committee. Release is subject to their approval and the appropriate safeguarding as determined by applicable legislation (GDPR and HIPAA).
